# Fibroblast growth factor-23 and cardiovascular disease among prevalent hemodialysis patients focusing on residual kidney function

**DOI:** 10.3389/fendo.2023.1099975

**Published:** 2023-07-11

**Authors:** Youn Kyung Kee, Hee Jung Jeon, Jieun Oh, Ajin Cho, Young-Ki Lee, Jong-Woo Yoon, Hyunsuk Kim, Tae-Hyun Yoo, Dong Ho Shin

**Affiliations:** ^1^ Department of Internal Medicine, Kangdong Sacred Heart Hospital, Hallym University, College of Medicine, Seoul, Republic of Korea; ^2^ Yonsei University College of Medicine, Seoul, Republic of Korea; ^3^ Department of Internal Medicine, Kangnam Sacred Heart Hospital, Hallym University College of Medicine, Seoul, Republic of Korea; ^4^ Department of Internal Medicine, Chuncheon Sacred Heart Hospital, Hallym University College of Medicine, Chuncheon, Gangwon-do, Republic of Korea

**Keywords:** fibroblast growth factor-23, cardiovascular disease, left ventricular diastolic dysfunction, hemodialysis patients, residual kidney function

## Abstract

**Background:**

In patients undergoing incident hemodialysis, increased fibroblast growth factor-23 (FGF-23) levels are associated with the development of cardiovascular disease (CVD), but the influence of residual kidney function (RFK) on this association is unclear. This study aimed to investigate the association between FGF-23 levels, RKF, and CVD in patients undergoing prevalent hemodialysis.

**Methods:**

This cross-sectional and longitudinal observational study included 296 patients undergoing maintenance hemodialysis for at least three months who were followed up for a median of 44 months. RKF was defined as 24-h urine output >200 mL, left ventricular (LV) diastolic dysfunction as E/E’ >15 on echocardiographic parameters. CVD was defined as hospitalization or emergency room visits due to cardiovascular causes, such as angina, myocardial infarction, or congestive heart failure.

**Results:**

The median intact FGF-23 (iFGF-23) level was 423.8 pg/mL (interquartile range, 171–1,443). Patients with an FGF-23 level > 423.8 pg/mL significantly had a lower proportion of RKF (39.2% vs. 60.1%, P < 0.001) and a higher proportion of LV diastolic dysfunction (54. 1% vs. 29.1%, P < 0.001) than those with an iFGF-23 level ≤ 423.8 pg/mL. The odds ratio (OR) for LV diastolic dysfunction was significantly higher in patients with RFK (OR per one-unit increase in the natural log-transformed iFGF-23 levels, 1.80; 95% confidence interval [CI]: 1.11–2.93) than in patients without RKF (OR per one-unit increase in the natural log-transformed iFGF-23 levels: 1.42; 95% CI: 1.01–1.99) in multivariate analysis (p < 0.001). During the follow-up period, 55 patients experienced CVD. The hazard ratio (HR) for CVD development was also significantly higher in patients with RKF (HR per one-unit increase in the natural log-transformed iFGF-23 levels, 2.64; 95% CI: 1.29–5.40) than those without RKF (HR per one-unit increase in the natural log-transformed iFGF-23 levels: 1.44; 95% CI: 1.04–1.99) in multivariate analysis (p = 0.05).

**Conclusions:**

Increased iFGF-23 levels were associated with LV diastolic dysfunction and CVD development in patients undergoing prevalent hemodialysis; however, the loss of RKF attenuated the magnitude of these associations. Therefore, in these patients, RKF strongly influenced the detrimental role of iFGF-23 in the development of CVD.

## 1 Introduction

Cardiovascular disease (CVD) is the leading cause of morbidity and mortality in patients with end-stage kidney disease (ESKD) undergoing hemodialysis (1, 2). Traditional risk factors for CVD, such as hypertension, diabetes, and dyslipidemia, do not fully account for the high prevalence of CVD in this population (3). Non-traditional risk factors for CVD, including vascular calcification, elevated asymmetrical dimethylarginine, anemia, volume overload, and others, contribute to the increased cardiovascular risk observed in patients with ESKD (4, 5). Fibroblast growth factor-23 (FGF-23), a hormone secreted by osteoblasts, plays an essential role in the development of CKD and MBD (6). In patients with CKD, serum FGF-23 levels gradually increase with decreasing kidney clearance. Increased FGF-23 levels help maintain a normal serum phosphate concentration range by enhancing urinary phosphate excretion and reducing phosphate absorption through decreased 1,25-dihydroxy vitamin D production (6). End-organ resistance to the phosphaturic action of FGF-23 due to a deficiency of Klotho, the required cofactor, may also increase FGF-23 levels (7). Usually, when these patients reach end-stage kidney disease (ESKD), FGE-23 levels can be up to 1,000-fold above the normal range (8).

Recent studies have found an association between elevated FGF-23 levels and CVD, including hypertension, LV hypertrophy, acute coronary syndrome, stroke, transient ischemic attack, and heart failure (9–13). In line with these findings, some observational studies on patients undergoing incident hemodialysis have shown that high FGF-23 levels are associated with a greater risk of CVD (14, 15). Interestingly, a few large-scale studies on patients undergoing prevalent hemodialysis also showed that high FGF-23 levels were associated with a greater risk of CVD (16, 17). However, the magnitude of this association was not as strong as that observed in patients undergoing incident hemodialysis. When hemodialysis is initiated, most patients with ESKD have substantial residual kidney function (RKF). Although these studies did not investigate RKF, the difference in the magnitude of this association might be related to RKF. Therefore, this study aimed to evaluate the association between increased FGF-23 levels and CVD development in patients undergoing prevalent hemodialysis, focusing on the differences in RKF.

## 2 Materials and methods

### 2.1 Ethics statement

The Institutional Review Boards of Kangam Sacred, Kangdong Sacred, and Chuncheon Sacred Heart Hospitals approved this study (Refs. 2018-03-19, 2018-07-01, 2018-96). This study was performed in accordance with the Declaration of Helsinki. All the patients provided written informed consent before enrolment in the study.

### 2.2 Patients

This study was conducted between January 2018 and January 2022 at three dialysis clinics: Kangam Sacred, Kangdong Sacred, and Chuncheon Sacred Heart Hospitals. Inclusion criterion was patients who had undergone hemodialysis for at least 3 months, three times a week for 4 h. Exclusion criteria were patients who were planning to transfer to other centers and those who did not undergo echocardiography.

### 2.3 Measurement of intact FGE-23 (iFGF-23)

Blood samples for iFGF-23, the full-length biological molecule, were collected in serum separator tubes, clotted at room temperature, centrifuged, frozen at -70 °C, and shipped on dry ice to Seoul Clinical Laboratories for measurement. The levels of iFGF-23 were measured using a commercial enzyme-linked immunosorbent assay kit (Kainos Laboratories Inc., Tokyo, Japan).

### 2.4 Data collection

Baseline characteristics, including demographic and clinical information, and biochemical parameters were collected from the medical records at the time of blood collection for iFGF-23 measurement. We conducted echocardiography within 1 week at baseline and calculated the mean interdialytic weight gain (IDWG) as an average of 10 values before baseline.

### 2.5 Echocardiographic measurements

This study used an ultrasound machine (Vivid 7; GE Vingmed Ultrasound AS, Horten, Norway) with a 2.5 MHz probe to perform comprehensive echocardiographic measurements based on the imaging protocols of the American Society of Echocardiography guidelines. This study calculated the LV ejection fraction and LV mass using modified biplane Simpson’s and Devereus and Reichek’s methods, respectively (18, 19). The LV mass index (LVMI) was calculated as follows: LVMI = LV mass/body surface area. This study assessed mitral inflow from an apical four-chamber view using Doppler echocardiography. Peak mitral in-flow velocities at early (E) and late (A) diastole and deceleration time were measured using mitral inflow profiles. The peak mitral annular velocities at early (E’) and late (A’) diastole were measured using Doppler tissue imaging.

### 2.6 Outcomes

Based on echocardiographic parameters, LV diastolic dysfunction was defined as E/E’ *>*15. Additionally, we designated CVD as an event requiring hospitalization or emergency room visits due to cardiovascular causes, such as angina, myocardial infarction, or congestive heart failure.

### 2.7 Statical analyses

We used descriptive statistics to compare baseline characteristics according to iFGF-23. Normally distributed variables were described as mean ± standard deviation and compared using the t-test for two groups. Nonnormally distributed variables were presented as a median and interquartile range, and compared using Mann–Whitney U test for the two groups. Categorical variables were described as frequencies and percentages, and compared using a chi-square test or Fisher’s exact test. Kaplan–Meier product estimation method was used to calculate the cumulative incidence of CVD according to the median iFGF-23 level. We used logistic regression to examine the association between iFGF-23 and LV diastolic dysfunction, and Cox proportional hazards analysis to investigate the association between iFGF-23 and CVD development. We used multivariate models to adjust for three models (1): demographic and clinical factors: age, sex, dialysis vintage, Kt/V, diabetes, and prior CVD (e.g., coronary artery disease and congestive heart failure); (2) markers of mineral metabolism: serum levels of calcium, phosphorus, and intact parathyroid hormone (iPTH); and (3) active vitamin D treatment: prescription of active or analog vitamin D. We analyzed the results of laboratory data on a continuous scale. Therefore, nonnormally distributed laboratory data (iPTH and iFGF-23 levels) were log-transformed. Hazard ratios (HR) or odds ratios (OR) and 95% confidence intervals (CI), corresponding to a one-unit increase in the natural log-transformed iFGF-23 levels, were provided. We also investigated the potential interactions of iFGF23 with RKF (defined as 24-h urine output >200 mL) for clinical outcomes. The correlation between mean IDWG and iFGF-23 was calculated using Pearson analysis. Statistical analyses were performed using SPSS 27.0 (SPSS Inc., Chicago, IL, USA).

## 3 Results

### 3.1 Study population

**Figure 1:**
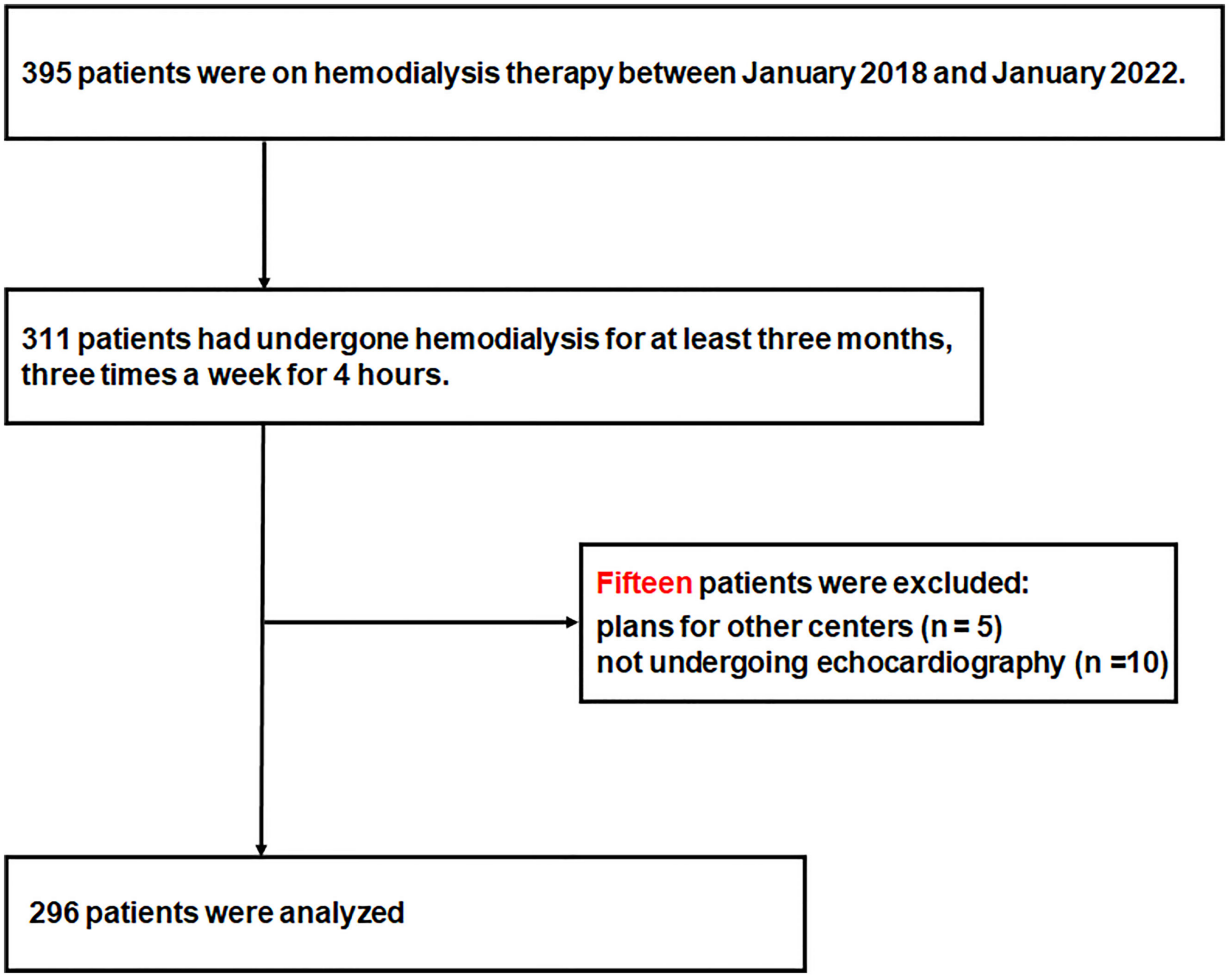
Flowchart of study population enrollment.

In total, 123, 140, and 132 patients were receiving hemodialysis therapy in Kangnam Sacred, Kangdong Sacred, and Chuncheon Sacred Heart Hospitals, respectively, between January 2018 and January 2019. Of these, 311 patients were eligible for inclusion in the study, and 15 patients were excluded because of plans of transfer to other centers (n=5) or because they did not undergo echocardiography (n=10). Thus, 296 patients were included in the study (**Figure 1**).

### 3.2 Baseline characteristics

**Figure 2:**
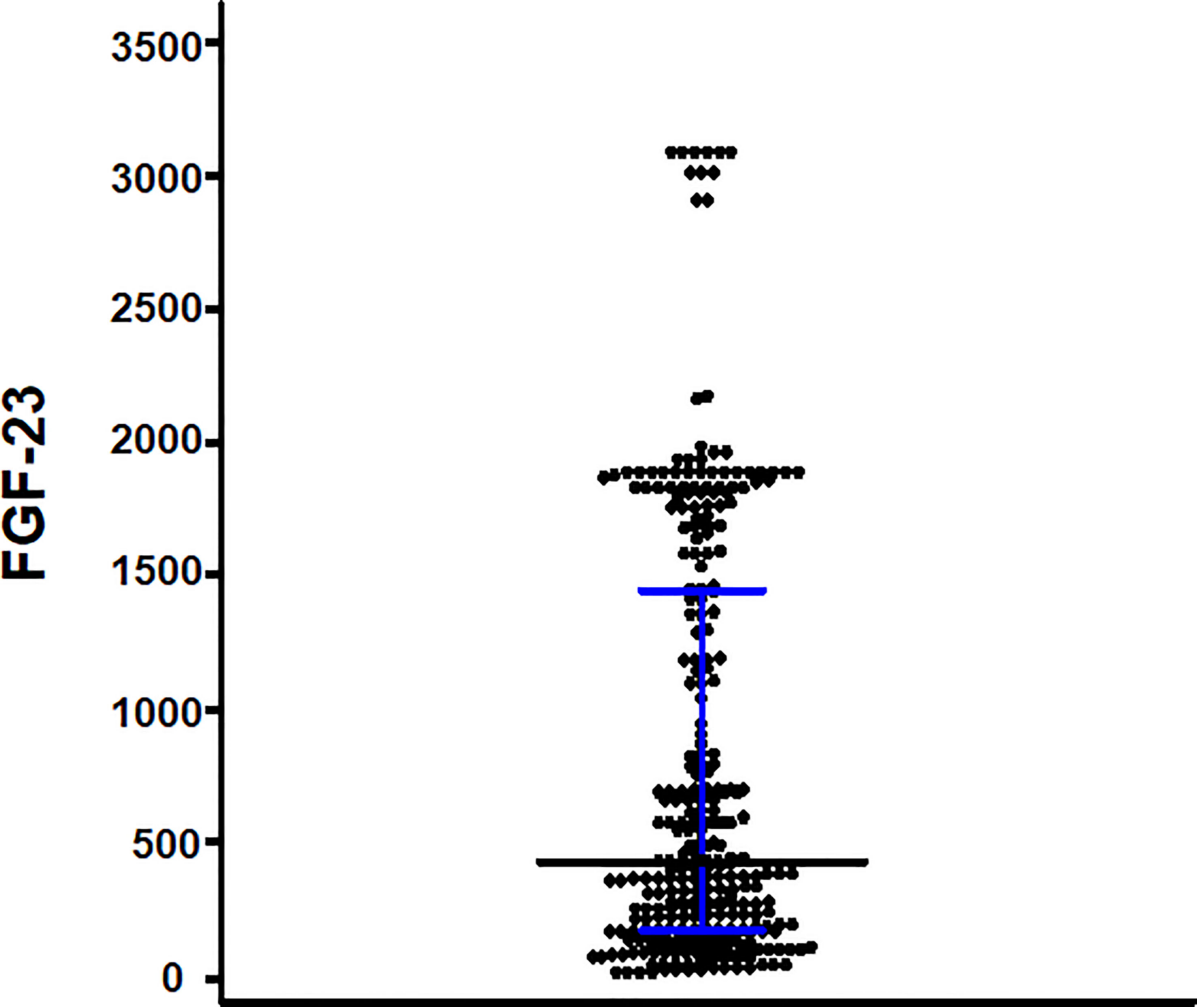
Scattered plots of the intact fibroblast growth factor-23 (iFGF-23) levels. The bar and error bars show the median and interquartile range.

Diabetes was the cause of ESKD in 173 (58.4%) patients. The median age was 63 years (interquartile range, 57–71 years), and 162 (54.7%) patients were men. The median iFGF-23 level was 423.8 pg/mL (interquartile range, 171.09–1,143.85 pg/mL) (**Figure 2**). Patients with iFGF-23 levels > 423.8 pg/mL had a longer hemodialysis vintage, lower proportion of RKF, greater mean IDWG, and were prescribed more noncalcium-based phosphate binders than those with iFGF-23 levels ≤423.8 pg/mL. Additionally, phosphate levels were higher in patients with iFGF-23 levels > 423.8 pg/mL (**Table 1**).

**Table 1:** Baseline characteristics, overall and according to intact FGF-23.

Variables	Total (n = 296)	iFGF-23 of ≤423.8 pg/mL (n=148)	iFGF-23 of >423.8 pg/mL (n=148)		P-value
Demographic data					
Age (years)	63 (57.0–71.0)	67 (58.5–74.0)	62.0 (56.0–69.0)		0.001
Male, n (%)	162 (54.7)	71 (48.0)	91 (61.5)		0.03
Clinical data					
Dialysis vintage (months)	42.6 (17.9–68.5)	40.4 (15.1–59.1)	44.6 (25.4 – 80.4)		0.02
Initial end-stage kidney disease					
Diabetes, n (%)	173 (58.4)	89 (60.1)	84 (56.8)		0.64
Non-diabetes, n (%)	123 (41.6)	59 (39.9)	64 (43.2)		
Previous cardiovascular disease, n (%)	103 (34.8)	49 (33.1)	54 (36.5)		0.54
Urine output ≥200 mL/d, n	147 (49.7)	89 (60.1)	58 (39.2)		0.001
Interdialytic weight gain	2.6 ± 0.8	2.5 ± 0.9	2.9 ± 0.7		0.001
Medication					
Calcium-based phosphate binders, n (%)	81 (27.4)	52 (35.1)	29 (19.6)		0.004
Non-calcium-based phosphate binders, n (%)	153 (51.7)	49 (33.1)	104 (70.3)		<0.001
Vitamin D analogs, n (%)	165 (55.7)	82 (55.4)	83 (56.1)		0.99
Cinacalcet, n (%)	42 (14.2)	16 (10.8)	26 (17.6)		0.13
Laboratory data					
Hemoglobin (g/dL)	10.3 ± 1.2	10.1 ± 1.0	10.4 ± 1.3		0.03
Albumin (g/dL)	3.8 ± 0.4	3.8 ± 0.3	3.9 ± 0.4		0.01
Calcium (mg/dL)	8.5 ± 0.7	8.4 ± 0.6	8.6 ± 0.7		0.08
Phosphate (mg/dL)	4.8 (4.0–5.9)	4.3 (3.5–5.1)	5.4 (4.6–6.3)		<0.001
iPTH (pg/mL)	239.8 (147.8–417.0)	226.3 (153.5–383.0)	279.9 (143.5–438.8)		0.17
Kt/V	1.7 ± 0.3	1.7 ± 0.3	1.6 ± 0.3		0.31

iFGF-23, intact fibroblast growth factor-23; iPTH, intact parathyroid hormone.

Values are expressed as median (interquartile range) or number (percentage).

### 3.3 Intact FGF-23 and LV diastolic dysfunction

**Table 2** presents the echocardiographic parameters of the two groups: patients with iFGF-23 levels ≤423.8 pg/mL and those with iFGF-23 levels > 423.8 pg/mL. The proportion of LV diastolic dysfunction was significantly higher in patients with iFGF-23 levels > 423.8 pg/mL than in those with iFGF-23 levels ≤ 423.8 pg/mL (**Table 2**). In univariate logistic regression analysis, increased iFGF-23 levels were significantly associated with LV diastolic dysfunction (OR per unit increase in the natural log-transformed iFGF-23 levels, 1.60; 95% CI, 1.30–1.95). After adjusting for model 1 covariates, increased iFGF-23 levels were significantly associated with LV diastolic dysfunction. Even after adjusting for models 2 and 3, increased iFGF-23 levels were significantly associated with LV diastolic dysfunction (**Table 3**).

**Table 2:** Echocardiac parameters in prevalent hemodialysis patients according to intact FGF-23.

Variables	Total (n=296)	iFGF-23 of ≤423.8 pg/mL (n = 148)	iFGF-23 of >423.8 pg/mL (n=148)	P-value
Echocardiac parameters				
LVEF (%)	57.2 ± 10.7	56.1 ± 11.9	58.4 ± 9.1	0.29
LVMI (g/m)	139.8 ± 41.3	142 ± 45.8	132 ± 34.4	0.06
E (cm/s)	78.5 ± 24.0	73.5 ± 23.2	84.6 ± 68.4	0.09
A (cm/s)	91.4 ± 21.1	91.2 ± 24.1	92.2 ± 30.2	0.79
E/A ratio	0.9 ± 0.4	0.8 ± 0.3	0.8 ± 0.5	0.01
E’/A’ ratio	0.6 ± 0.2	0.6 ± 0.4	0.8 ± 1.3	0.19
E/E’ ratio	18.9 ± 9.1	15.2 ± 5.9	17.1 ± 8.6	0.02
Diastolic dysfunction, n (%)	123 (41.6)	43 (29.1)	80 (54.1)	<0.001
DT	216.3 ± 67.9	223.1 ± 72.9	213.7 ± 67.8	0.30

iFGF-23, intact fibroblast growth factor-23; LVEF, left ventricular ejection fraction; LVMI, left ventricular mass index; DT, deceleration time.

Values are expressed as mean ± standard deviation.

**Table 3:** Associations of serum intact FGF-23 with left ventricular diastolic dysfunction and the interaction of intact FGF-23 with residual kidney function.

	Overall (n=296)	Urine output ≥200 mL/dL (n=147)	Urine output <200 mL/dL (n=149)	P for interaction
Median FGF-23 (IQR), pg/mL	423.8 (171.1–1443.9)	321.62 (135.41–681.11)	690.33 (257.08–1778.50)	
^*^OR (95% CI) for diastolic dysfunction				
No adjustment	1.60 (1.30–1.95)	1.59 (1.13–2.52)	1.39 (1.07–1.79)	<0.001
Model 1	1.54 (1.23–1.94)	1.99 (1.28–3.11)	1.38 (1.04–1.84)	<0.001
Model 2	1.51 (1.17–1.95)	1.51 (1.17–1.95)	1.41 (1.02–1.95)	<0.001
Model 3	1.50 (1.16–1.94)	1.87 (1.11–3.14)	1.41 (1.01–1.95)	<0.001

FGF-23, fibroblast growth factor-23; OR, odds ratio; CI, confidence interval; IQR, interquartile range; 1-SD, one-standard deviation

^*^ORs are per unit increase in natural log-transformed intact FGF-23 levels

Model 1 was adjusted for age, sex, diabetes, dialysis vintage, prior cardiovascular disease, interdialytic weight gain, and Kt/V.

Model 2 was adjusted for model 1 covariates plus calcium, phosphate, and natural log-transformed intact parathyroid hormone levels.

Model 3 adjusted for model 2 covariates plus vitamin D analogs treatment.

### 3.4 Intact FGF-23 and CVD

**Figure 3:**
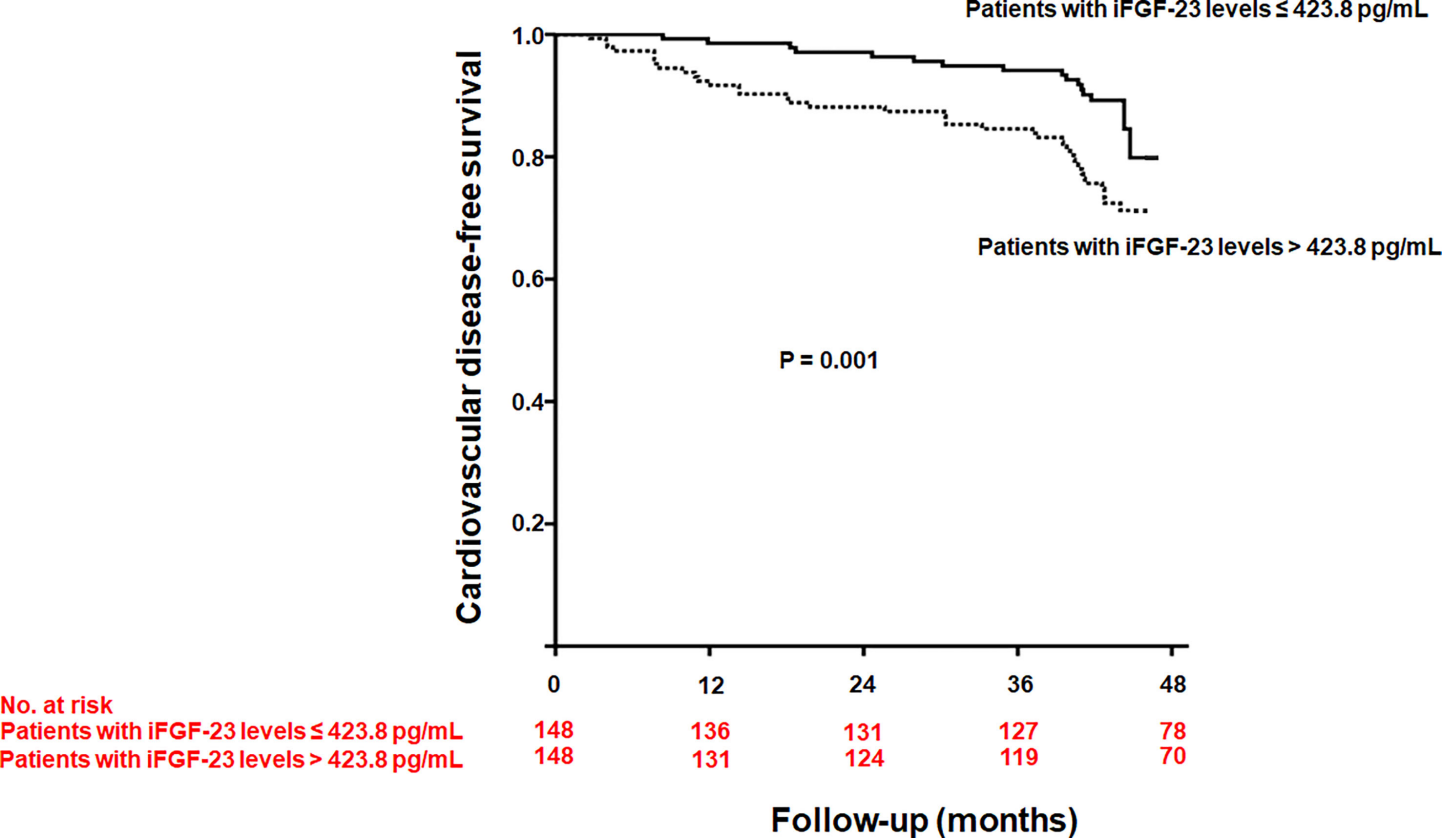
Kaplan-Meier curve of developing cardiovascular disease according to the median intact fibroblast growth factor-23 (iFGF-23) levels (*P* = 0.001).

During a median follow-up of 44 months (interquartile range, 41.0–44.6 months), 55 patients experienced CVD, with 17 cases of angina, 15 cases of myocardial infarction, and 23 cases of congestive heart failure. Cardiovascular disease was significantly higher in patients with iFGF-23 levels > 423.8 pg/mL than in those with iFGF-23 levels ≤ 423.8 pg/mL (26.4% vs. 10.8%, P = 0.001) (**Figure 2**). The cumulative probability of incident CVD was also significantly higher in patients with iFGF-23 levels > 423.8 pg/mL than in those with iFGF-23 levels ≤ 423.8 pg/mL (**Figure 3**). In the univariate Cox regression analysis, increased iFGF-23 levels were significantly associated with the development of CVD. Additionally, increased iFGF-23 levels were significantly associated with the development of CVD, even when the analysis was adjusted for models 1, 2, and 3 (**Table 4**).

**Table 4:** Associations of serum intact FGF-23 with cardiovascular diseases and the interaction of intact FGF-23 with residual kidney function.

	Overall (n=296)	Urine output ≥200 mL/dL (n=147)	Urine output <200 mL/dL (n=149)	P for interaction
Median FGF-23 (IQR), pg/mL	423.8 (171.1–1443.9)	321.62 (135.41–681.11)	690.33 (257.08–1778.50)	
^*^HR (95% CI) for cardiovascular disease				
No adjustment	1.58 (1.24–2.02)	2.27 (1.36–3.80)	1.24 (0.95–1.61)	0.06
Model 1	1.52 (1.17–1.98)	2.84 (1.41–5.75)	1.33 (1.00–1.78)	0.06
Model 2	1.58 (1.17–2.13)	3.02 (1.37–6.67)	1.45 (1.05–2.02)	0.04
Model 3	1.58 (1.17–2.13)	3.17 (1.38–7.29)	1.45 (1.05–2.02)	0.04

FGF-23, fibroblast growth factor-23; HR, hazard ratio; CI, confidence interval; IQR, interquartile range.

^*^HRs are per unit increase in natural log-transformed iFGF-23.

Model 1 was adjusted for age, sex, diabetes, dialysis vintage, prior cardiovascular disease, interdialytic weight gain, and Kt/V.

Model 2 was adjusted for model 1 covariates and calcium, phosphate, and natural log-transformed parathyroid hormone levels.

Model 3 adjusted for model 2 covariates plus vitamin D analogs treatment.

### 3.5 Interaction with RKF

Patients with RKF had lower iFGF-23 levels than those without RKF. However, the OR for LV diastolic dysfunction (OR per one-unit increase in the natural log-transformed iFGF-23 levels, 1.59; 95% CI: 1.13–2.52) in patients with RKF was more robust than that in patients without RKF (OR per one-unit increase in the natural log-transformed iFGF-23, 1.39; 95% CI: 1.07–1.79; p < 0.001). The higher magnitude of OR for LV diastolic dysfunction in patients with RKF than in patients without RKF was also maintained significantly by further adjustments for models 1, 2, and 3 (**Table 3**). Interestingly, the HR for developing CVD was significantly higher in patients with RKF than in patients without RKF when the analysis was adjusted for models 2 and 3.

### 3.6 Intact FGF-23, IDWG, and RKF

Although the mean IDWG was positively correlated with iFGF-23 levels in patients with RKF (r = 0.24, p = 0.004), there was no correlation between the mean IDWG and iFGF-23 levels in patients without RKF (r = -0.27, p = 0.74). However, in patients without RKF, the high mean IDWG was significantly associated with an increased risk of LV diastolic dysfunction and CVD development in the univariate and multivariate analyses (**Supplemental Tables 1, 2**).

## 4 Discussion

This cross-sectional and longitudinal observational study confirmed that increased iFGF-23 levels were significantly associated with LV diastolic dysfunction in patients undergoing prevalent hemodialysis. Additionally, increased iFGF-23 levels are significantly associated with the development of CVD. However, the loss of RKF attenuated the magnitude of these associations, even though the median iFGF-23 levels were higher in patients without RKF than in those with RKF.

Preservation of RKF has long been recognized as a significant beneficial factor in cardiovascular and overall mortality in hemodialysis patients. Even a minimal level of RKF has been associated with better fluid and electrolyte balance, enhanced clearance of middle molecules, lower inflammation levels, and improved quality of life (20, 21). Thus, understanding the role of RKF in mitigating the detrimental effects of FGF-23 on CVD is essential to developing potential therapeutic strategies for this patient population.

Observational cohort studies have shown a strong association between increased FGF-23 levels and the development of CVD in patients undergoing incident hemodialysis. These studies divided patients into three or four groups according to FGF-23 levels and demonstrated HRs of developing CVD for the highest FGF-23 level group versus the lowest FGF-23 level group, which ranged between 3–5 (14, 15). Meanwhile, in cohort studies on patients undergoing prevalent hemodialysis, HRs of developing CVD for the highest FGF-23 level group versus the lowest FGF-23 level group ranged between 1–1.2 (16, 17). Interestingly, a Japanese cohort study showed that long-term dialysis attenuated the association between increased FGF-23 levels and CVD development (22). Therefore, differences in the magnitude of this association might be related to differences in baseline characteristics between patients enrolled in the study, and RKF may be one of the differences. To understand these findings, we first evaluated the association between increased iFGF-23 levels and LV diastolic dysfunction in patients undergoing prevalent hemodialysis, focusing on differences in RFK.

Previous studies have indicated that LV diastolic dysfunction is associated with CVD, and is an independent predictor of CVD development in patients (23–25). Therefore, investigating LV diastolic dysfunction in these patients will help understand the association between increased FGF-23 levels and CVD development. Although several observational clinical studies showing that increased FGF-23 levels were associated with LV diastolic dysfunction cannot prove causality (26–29), *in vitro* and experimental animal studies have demonstrated that FGF-23 induced LV hypertrophy is related to LV diastolic dysfunction (11). FGF-23 causes pathological hypertrophy of isolated rat cardiomyocytes *via* FGF receptor-dependent activation of the calcineurin-nuclear factor of activated T cells signaling pathway, but this effect is independent of Klotho (11). These findings suggest that the pathogenesis of FGF-23-induced LV hypertrophy may be ongoing in patients undergoing hemodialysis, thereby promoting LV diastolic dysfunction. However, in this study, the magnitude of the association between increased iFGF-23 levels and LV diastolic dysfunction in patients without RKF was not as strong as that in those with RKF. Although iFGF-23 is associated with LV diastolic dysfunction, age, sex, diabetes, dialysis vintage, prior CVD, calcium, phosphate, and iPTH play significant roles in inducing LV diastolic dysfunction (30–35). Especially mechanical stress, such as volume overload, causes LV hypertrophy as the primary maladaptive response (36). Persistent fluid overload is commonly observed in patients with ESKD without RKF. In our study, the high mean IDWG was significantly associated with the risk of LV diastolic dysfunction in patients without RKF. Therefore, in patients without RKF, IDWG may diminish the effect of increased iFGF-23 levels on LV diastolic dysfunction.

In recent years, it has become clear that FGF-23 directly influences calcium and sodium handling in the distal nephron of the kidney. In the distal tubular epithelium, FGF-23 regulates the apical membrane abundance of epithelial transient receptor potential vanilloid-5 and sodium-chloride cotransporter (9). In our study, iFGF 23 and mean IDWG were positively correlated in patients with RKF but not significantly in patients without RKF. This is probably because the direct effect of increased FGF-23 on the distal nephron of the kidney resulted in sodium retention and blood volume expansion in patients with RKF. Therefore, the absence of RKF would reduce the effect of increased FGF-23 levels on LV diastolic dysfunction. Another possible explanation for how the loss of RKF reduces the effect of elevated FGF-23 levels on left ventricular diastolic dysfunction is that it leads to decreased levels of certain hormones that contribute to the detrimental effects of FGF-23 on the cardiovascular system. For example, a study has suggested that the renin-angiotensin-aldosterone system (RAAS) may play a role in the adverse effects of FGF-23 on the heart and that the attenuation of these effects with the loss of RKF may be due to a decrease in RAAS activity (37). However, it is essential to remember that these are just hypotheses and that other mechanisms may also be involved. The exact mechanism of this attenuating effect has yet to be fully explored and requires further investigation.

Therefore, RKF plays a pivotal role in the detrimental effects of increased FGF-23 levels on the development of CVD. FGF-23 is an approximately 32-kDa glycoprotein with N- and C-terminal regions (38). There are two main types of assays for measuring FGF-23 levels in humans. The iFGF-23 assay binds two epitopes that flank the proteolytic cleavage site, presumably detecting only biologically active, full-length FEG-23 (~32 kDa) (39). In contrast, the C-terminal FGF-23 (cFGF-23) assay binds to epitopes within the C-terminal region of the FGF-23 protein, and detects both full-length and processed C-terminal fragments (~14kDa) (40). Based on the idea that the iFGF-23 assay might be superior because it detects the full-length FGF23 molecule and not a mixture of full-length FGF23 and degradation products (41), we used the iFGF-23 assay in this study. However, most prior studies have reported associations between FGF-23 and clinical outcomes using the cFGF-23 assay (14, 15, 42, 43). Additionally, a few studies have shown that the iFGF-23 assay showed much weaker associations with similar endpoints than the cFGF-23 assay (44, 45). Therefore, in our study, the association between increased FGF-23 levels and clinical outcomes may have been weakened because of the usage of an iFGF-23 assay. Nevertheless, we showed the importance of RKF by showing that the loss of RKF attenuated the magnitude of the associations between increased iFGF-23 and LV diastolic dysfunction and CVD development.

Our study had several limitations. First, this was a small-scale observational study. Second, we did not measure cFGF-23 levels, which might have provided additional information on the regulation of FGF-23. Third, we could not determine whether the association between increased iFGF-23 levels and LV diastolic dysfunction is causal. Lastly, there were numerous potential confounders that could affect the relationship between iFGF-23 levels, RKF, and CVD. Although we attempted to account for several of these factors in our multivariable model, there may be other unmeasured confounding factors that we could not adjust for.

In conclusion, we showed that increased iFGF-23 levels are associated with LV diastolic dysfunction and CVD development in patients undergoing prevalent hemodialysis. The loss of RKF attenuated the magnitude of these associations, highlighting the strong influence of RKF on the detrimental role of iFGF-23 in the development of CVD. Nonetheless, the observed associations between iFGF-23 levels, RKF, and CVD should be interpreted with caution, as there may be other unmeasured confounding factors. Further large-scale studies are needed to understand the complex relationship between these factors fully. If confirmed, FGF–receptor blocker treatment could be considered in patients undergoing hemodialysis with RKF to prevent CVD development.

## Data availability statement

The original contributions presented in the study are included in the article/**Supplementary Material**. Further inquiries can be directed to the corresponding author.

## Ethics statement

The studies involving human participants were reviewed and approved by the Institutional Review Boards of Kangam Sacred, Kangdong Sacred, and Chuncheon Sacred Heart Hospitals. The patients/participants provided their written informed consent to participate in this study.

## Author contributions

DHS conceived and designed the study. YKK, HJJ, YKL, AC., JWY, and HK acquired data. DHS, AC, and YKK analyzed and interpreted the data. DHS and YKK wrote the paper. DHS, YKL, and JWY reviewed the manuscript for important intellectual content and approved the final version. During the revision, THY provided invaluable advice and assistance in addressing the reviewers' comments and concerns.
