# Supplementary material for: Fibroblast growth factor-23 and cardiovascular disease among prevalent hemodialysis patients focusing on residual kidney function
Source: Front Endocrinol (Lausanne). 2023 Jul 11;14:1099975. doi: 10.3389/fendo.2023.1099975 (PMC10368752; doi:10.3389/fendo.2023.1099975)
Supplement: Supplementary file 1 [file Table_1.docx]

Supplementary Material

Supplementary Tables

Supplement Table 1. Factors related to left ventricular diastolic dysfunction according to residual kidney function.

|  | Unadjusted |  | Adjusted |  |  |  |  |  |
| --- | --- | --- | --- | --- | --- | --- | --- | --- |
|  |  |  | Overall  (n=296) |  | Urine output ≥200 mL/dL  (n=147) |  | Urine output <200 mL/dL  (n=149) |  |
|  | OR (95% CI) | P-value | OR (95% CI) | P-value | OR (95% CI) | P-value | OR (95% CI) | P-value |
| Age | 0.99 (0.96–1.02) | 0.37 | 0.99 (0.95–1.02) | 0.43 | 1.01 (0.96–1.05) | 0.74 | 0.99 (0.95–1.02) | 0.43 |
| Male (vs. female) | 0.83 (0.52–1.32) | 0.43 | 1.29 (0.52–3.21) | 0.59 | 0.57 (0.20–1.62) | 0.30 | 1.29 (0.52–3.21) | 0.59 |
| Diabetes (vs. non-diabetes) | 1.91 (1.18–3.10) | 0.01 | 2.23 (1.06–4.66) | 0.03 | 2.59 (0.94–7.11) | 0.07 | 2.23 (1.06–4.66) | 0.03 |
| Prior CVD (vs. non-prior CVD) | 1.26 (0.78–2.04) | 0.35 | 1.02 (0.48–2.18) | 0.96 | 1.61 (0.63–4.14) | 0.33 | 1.02 (0.48–2.18) | 0.96 |
| Dialysis vintage (per month) | 1.01 (1.00–1.01) | 0.01 | 1.00 (0.99–1.01) | 0.87 | 1.00 (0.98–1.02) | 0.99 | 1.00 (0.99–1.01) | 0.87 |
| Mean IDWG (per 1 kg) | 1.98 (1.44–2.72) | <0.001 | 2.50 (1.32–4.72) | 0.005 | 0.61 (0.33–1.12) | 0.11 | 2.50 (1.32–4.72) | 0.01 |
| Kt/V (per 0.1) | 1.02 (0.95–1.10) | 0.60 | 1.07 (0.93–1.23) | 0.37 | 0.86 (0.70–1.07) | 0.17 | 1.07 (0.93–1.23) | 0.37 |
| Serum calcium (per 1 mg/dL) | 1.28 (0.91–1.79) | 0.16 | 0.90 (0.53–1.52) | 0.69 | 1.10 (0.53–2.31) | 0.80 | 0.90 (0.53–1.52) | 0.69 |
| Serum phosphate (per 1 mg/dL) | 1.24 (1.05–1.47) | 0.01 | 0.99 (0.74–1.33) | 0.96 | 1.04 (0.70–1.53) | 0.85 | 0.99 (0.74–1.33) | 0.96 |
| ^*^iPTH (per one unit increase) | 1.04 (0.81–1.35) | 0.76 | 1.04 (0.69–1.57) | 0.87 | 0.76 (0.44–1.30) | 0.31 | 1.04 (0.69–1.57) | 0.87 |
| ^*^iFGF-23 (per one unit increase) | 1.60 (1.30–1.95) | <0.001 | 1.41 (1.01–1.95) | 0.04 | 1.87 (1.11–3.14) | 0.02 | 1.41 (1.01–1.95) | 0.04 |
| Vitamin D analogs treatment (vs. non-vitamin D analogs treatment) | 1.03 (0.64–1.63) | 0.92 | 1.12 (0.50–2.47) | 0.79 | 1.44 (0.54–3.84) | 0.47 | 1.12 (0.50–2.47) | 0.79 |

OR, odds ratio; CI, confidence interval; CVD, cardiovascular disease; IDWG, interdialytic weight gain; iPTH, intact parathyroid hormone; iFGF-23, intact fibroblast growth factor-23

*Natural log scale

Supplement Table 2. Factors related to cardiovascular disease according to residual kidney function

|  | Unadjusted |  | Adjusted |  |  |  |  |  |
| --- | --- | --- | --- | --- | --- | --- | --- | --- |
|  |  |  | Overall  (n=296) |  | Urine output ≥200 mL/dL  (n=147) |  | Urine output <200 mL/dL  (n=149) |  |
|  | HR (95% CI) | P-value | HR (95% CI) | P-value | HR (95% CI) | P-value | HR (95% CI) | P-value |
| Age | 1.00 (0.97–1.02) | 0.75 | 1.00 (0.97–1.04) | 0.77 | 0.96 (0.90– 1.02) | 0.16 | 1.04 (1.00–1.08) | 0.03 |
| Male (vs. female) | 1.19 (0.70–2.03) | 0.52 | 1.12 (0.58–2.15) | 0.74 | 0.62 (0.15–2.54) | 0.51 | 1.99 (0.90–4.43) | 0.09 |
| Diabetes (vs. non-diabetes) | 0.76 (0.45–1.28) | 0.30 | 0.85 (0.49–1.46) | 0.55 | 1.03 (0.26–4.16) | 0.97 | 0.80 (0.41–1.54) | 0.50 |
| Prior CVD (vs. non-prior CVD) | 1.08 (0.62–1.88) | 0.78 | 0.88 (0.48–1.59) | 0.66 | 0.90 (0.24–3.33) | 0.87 | 0.69 (0.33–1.41) | 0.31 |
| Dialysis vintage (per month) | 1.00 (1.00–1.01) | 0.59 | 1.00 (0.99–1.01) | 0.83 | 0.97 (0.93–1.01) | 0.11 | 1.00 (0.99–1.01) | 0.97 |
| Mean IDWG (per 1 kg) | 1.49 (1.08–2.06) | 0.02 | 1.30 (0.88–1.92) | 0.19 | 0.44 (0.19–1.00) | 0.05 | 1.73 (1.00–2.97) | 0.05 |
| Kt/V (per 0.1) | 1.00 (0.91-1.09) | 0.88 | 0.98 (0.88-1.09) | 0.68 | 0.78 (0.55-1.11) | 0.17 | 1.01 (0.90-1.13) | 0.86 |
| Serum calcium (per 1 mg/dL) | 1.52 (1.05–2.19) | 0.03 | 1.20 (0.82–1.77) | 0.35 | 1.31(0.51–3.40) | 0.58 | 0.94 (0.60–1.48) | 0.80 |
| Serum phosphate (per 1 mg/dL) | 1.08 (0.89–1.32) | 0.45 | 0.90 (0.70–1.15) | 0.39 | 0.68 (0.39–1.17) | 0.16 | 0.82 (0.60–1.11) | 0.20 |
| ^*^iPTH (per one unit increase) | 0.79 (0.60–1.04) | 0.09 | 0.83 (0.62–1.11) | 0.21 | 0.81 (0.42–1.56) | 0.53 | 0.78 (0.54–1.11) | 0.16 |
| ^*^iFGF-23 (per one unit increase) | 1.58 (1.24–2.02) | <0.001 | 1.58 (1.17–2.13) | 0.003 | 3.17 (1.38–7.29) | 0.007 | 1.45 (1.05–2.02) | 0.03 |
| Vitamin D analogs treatment (vs. non-vitamin D analogs treatment) | 0.95 (0.56–1.62) | 0.85 | 1.04 (0.58–1.87) | 0.90 | 1.44 (0.33–6.28) | 0.63 | 1.01 (0.47–2.18) | 0.98 |

HR, hazard ratio; CI, confidence interval; CVD, cardiovascular disease; IDWG, interdialytic weight gain; iPTH, intact parathyroid hormone; iFGF-23, intact fibroblast growth factor-23

*Natural log scale
